# Mixed neuroendocrine-non-neuroendocrine neoplasm of the gallbladder: case report and literature review

**DOI:** 10.1186/s13000-022-01231-6

**Published:** 2022-06-17

**Authors:** Xu Ren, Hong Jiang, Kan Sun, Xufu Qin, Yongping Qu, Tian Xia, Yan Chen

**Affiliations:** 1 Digestive Hospital of Heilongjiang Provincial Hospital Affiliated to Harbin University of Technology, No. 405, Guogeli Street, Harbin, 150001 Heilongjiang China; 2 Department of Pathology, Heilongjiang Provincial Hospital Affiliated to Harbin University of Technology, Harbin, 150001 Heilongjiang Province China; 3 Department of General Surgery, Heilongjiang Provincial Hospital Affiliated to Harbin University of Technology, Harbin, 150001 Heilongjiang Province China; 4 Department of Gastroenterology, Heilongjiang Provincial Hospital Affiliated to Harbin University of Technology, Harbin, 150001 Heilongjiang Province China; 5 Digestive Endoscopy Center, Heilongjiang Provincial Hospital of Harbin Institute of Technology, Harbin, 150001 Heilongjiang Province China; 6 Hospital Information Center of Heilongjiang Province Affiliated to Harbin Institute of Technology, Harbin, 150001 Heilongjiang Province China

**Keywords:** Mixed neuroendocrine–non-neuroendocrine neoplasm, Large cell neuroendocrine carcinoma, Papillary adenocarcinoma, Gallbladder, Median survival time, Case report

## Abstract

**Background:**

Mixed neuroendocrine–non-neuroendocrine neoplasms (MiNENs) of the gallbladder are rare malignancies. Here we presented two cases and reviewed the related literature.

**Case presentation:**

Our two patients were postoperatively diagnosed with gallbladder MiNENs, which pathologically consisted of a large cell neuroendocrine carcinoma and papillary adenocarcinoma. After cholecystectomy, one patient had a survival time of 30 months, while the other remained alive through 12 months of follow-up. In the literature, a total of 72 cases of gallbladder MiNENs were identified, and with our two patients included, we calculated a male-to-female ratio of 0.22 and a mean age of 64.5 years for the 74 reported cases. About one-half of these patients were found to have gallstones and presented with abdominal pain or discomfort in a relatively early stage. The preoperative diagnosis of these 74 cases mainly relied on abdominal ultrasound, contrast-enhanced computed tomography (CT) scanning, and magnetic resonance imaging or positron emission tomography/CT. However, the final diagnosis was established based upon the pathological evidence and expression of synaptophysin (Syn) and/or chromogranin A identified by immunohistochemical staining or neurosecretory granules detected by electron microscopy. Fifty-eight patients (78.4%) underwent various operations including simple cholecystectomy (*n* = 14), en bloc cholecystectomy (*n* = 9), standard or non-standard radical cholecystectomy (*n* = 25), or extended radical cholecystectomy (*n* = 6). The mean size of the resected gallbladder masses was 50.8 ± 36.1 mm (*n* = 63) with regional lymph node metastasis in 37 patients (52.1%), liver invasion or staging greater than T3 in 33 patients (45.8%), and hepatic metastasis in 26 patients (35.1%). The postoperative median survival time was 36 ± 11.42 months (95% confidence interval, 13.62 to 58.38 months). The log-rank analysis did not find that postoperative adjuvant chemotherapy contributed to a longer survival time relative to that among the patients who did not receive chemotherapy (numbers of patients, 15 versus 43; survival times, 36 months versus 30 months, *p* > 0.05).

**Conclusions:**

Our two cases and the cases in the literature suggest that MiNENs of the gallbladder predominantly occur in women; are associated with early lymph node metastasis, local hepatic invasion, and hepatic metastasis; and can be managed by various surgeries as well as chemotherapy combined with somatostatin analogs.

## Background

As an extremely rare pathological entity, mixed neuroendocrine–non-neuroendocrine neoplasms (MiNENs) pose inherent diagnostic and management challenges [1]. Based on the statistical results from Europe, the incidence of MiNENs is less than 0.01/100,000 cases per annum, and the common sites of origin of MiNENs are, in descending order, the appendix (60.3%), colon-rectum (14.5%), and rarely biliary tract (1.6%) [2], and two-thirds of cases in the biliary tract primarily arise from the gallbladder [3].

Neuroendocrine carcinomas (NECs) of the gallbladder only account for 4% of all malignant gallbladder neoplasma, and more than one-third of diagnosed gallbladder NECs coexist with an adenocarcinoma component (MiNENs) [1]. Clinically, MiNENs of the gallbladder that present as either cholelithiasis or gallbladder neoplasms have an insidious onset, are difficult to diagnose early, show rapid progression, and are associated with short survival time. Pathologically, MiNENs of the gallbladder generally are epithelial neoplasms but possess mixed pathophysiological natures of both a neuroendocrine neoplasm and adenocarcinoma, which are found to be more highly aggressive than gallbladder NEC alone in terms of regional lymph node and hepatic metastases [4]. This is partly attributed to the delay in their diagnosis and treatment [3], resulting in enhanced malignancy and a diminished long-term prognosis.

Our understanding of gallbladder MiNENs has been restricted by the rarity of this neoplasm and the limited amount of published data. Therefore, we reviewed the literature along with our case presentation to provide more information for improving the understanding of this disease to achieve early diagnosis and treatment.

### Case presentation

#### Case one

**Fig. 1:**
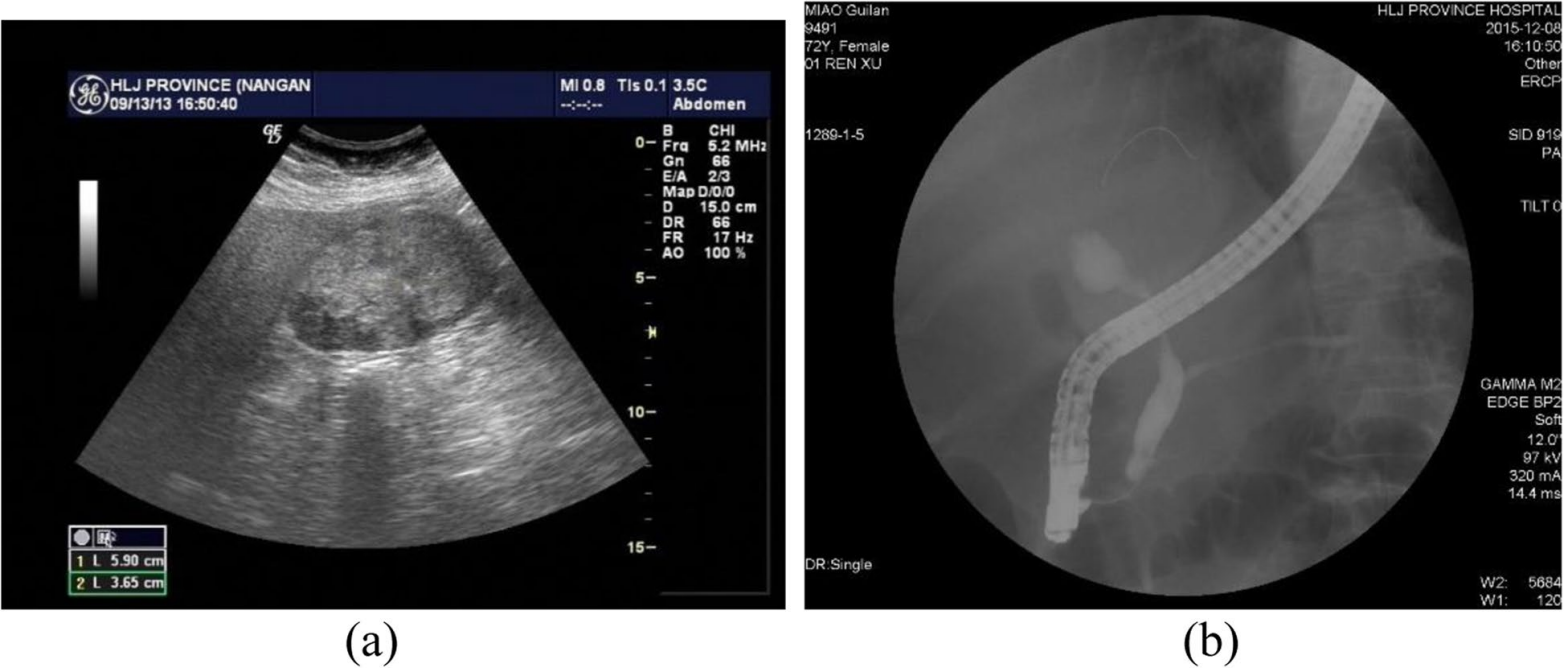
Case 1: **a** abdominal ultrasonography. A 6-cm-diameter, irregular polypoid mass was visualized in the gallbladder lumen. **b** ERCP revealed common hepatic duct stricture 27 months after the procedure, and a bile duct biopsy was performed

A 70-year-old female patient with right upper abdominal pain for 4 days was admitted to our hospital on September 15, 2013, with gallstones and suspected gallbladder cancer. Despite a normal CA19–9 and neuron-specific enolase (NSE) level, the carcinoembryonic antigen (CEA) and alpha-fetoprotein (AFP) levels were elevated at 8.04 ng/ml (normal: < 4.0 ng/ml) and 55.2 ng/ml (normal: 0.89 to 8.78 ng/ml), respectively. Abdominal ultrasound showed a 6-cm sized mass with an irregular and heterogeneous echogenicity (Fig. 1a) and a stone in the gallbladder. Contrast-enhanced computed tomography (CT) scanning and magnetic resonance imaging (MRI) further identified an enhanced gallbladder mass. Subsequently, open cholecystectomy was performed without major adverse events, during which hepatic metastases were not observed, but multiple enlarged lymph nodes in the hepatoduodenal ligament were found to infiltrate the portal vein and could not be dissected. Two years later, the patient was re-admitted on December 7, 2015, for obstructive jaundice and hepatic metastasis. The patient subsequently underwent endoscopic retrograde cholangiopancreatography (ERCP), in which a stricture in the middle extrahepatic bile duct (Fig. 1b) was identified and further managed with biliary stenting. Three months later, the patient died from systemic organ failure, with a survival time of 30 months. A lesson from this case is that any large size gallbladder lesions should be further investigated considering the possibility of MiNENs.

**Fig. 2:**
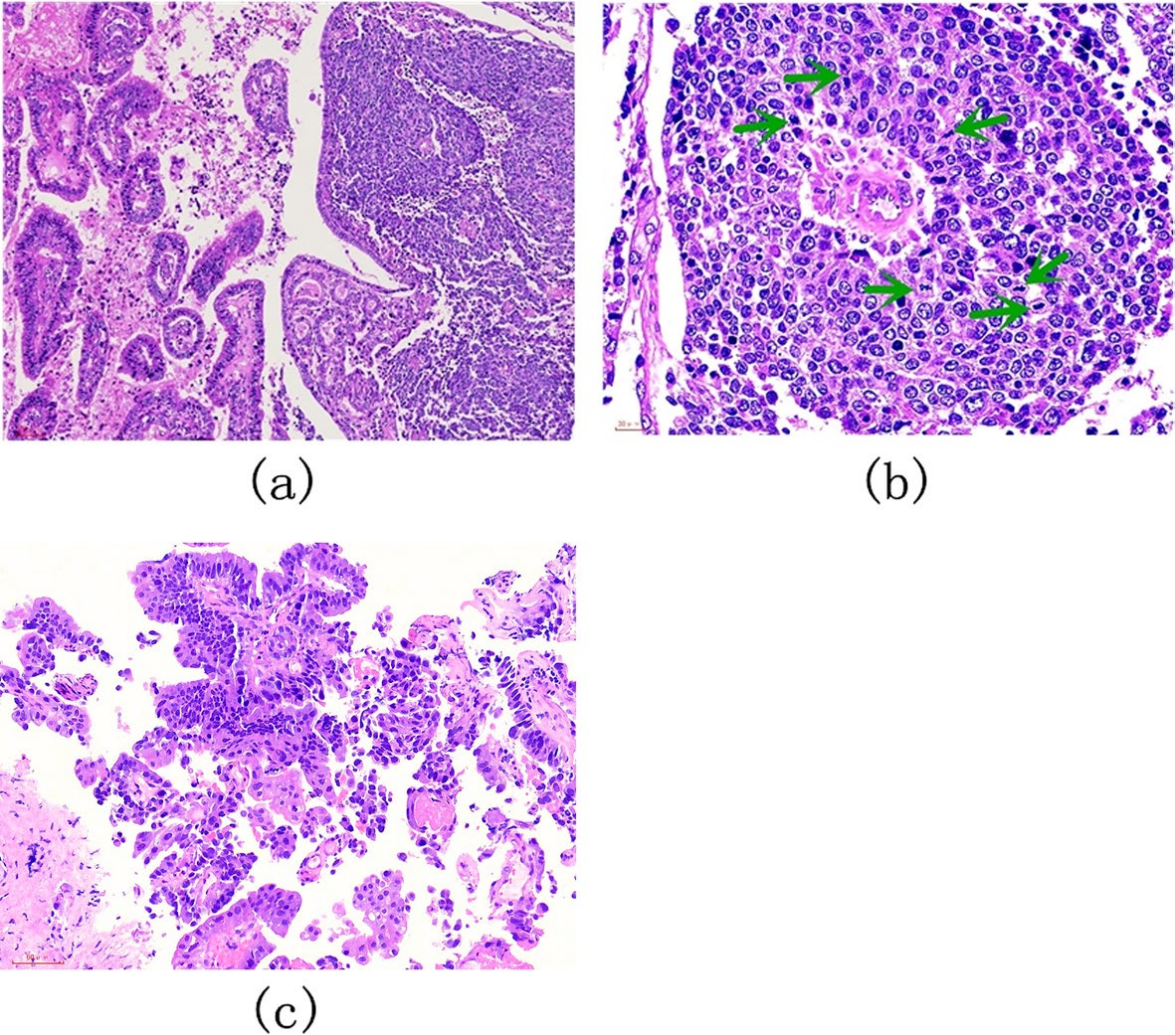
Case 1: Histological findings of MiNEN in the gallbladder by hematoxylin and eosin (HE) staining. **a** Two components, LCNEC (right) and papillary adenocarcinoma (left) showed mixed composition and solid sheet distribution, with an obvious transitional zone between the two tissues. Magnification, × 100. **b** large cells arranged in solid sheets, with vesicular nuclei and abundant eosinophilic cytoplasm, and tumor cells had large-sized densely stained round to oval nuclei, in some cells visible nucleoli, high mitotic index (arrows), and focal tumoral necrosis consistent with LCNEC were showed. Magnification, × 400. **c** Metastatic lesion of the bile duct showing well-differentiated papillary adenocarcinoma. Magnification, × 100

The gross findings of the incised gallbladder, in this case, showed a 70 mm × 50 mm soft polypoid mass in the neck and body, and a 2-cm stone in the gallbladder. Histopathological examination showed about 65% large cell neuroendocrine carcinoma (LCNEC) and 35% moderately differentiated papillary adenocarcinoma in the pathological sections with a distinct transitional zone between the two components (Fig. 2a). Large cells with a high mitotic rate (60 mitoses/2 mm^2^) were found in solid sheets or organoid nests, and also other microscopic characteristics of LCNEC were observed (Fig. 2b). Additionally, cancer emboli were observed in the lymphatic vessels. Meanwhile, LCNEC invaded the gallbladder, while papillary adenocarcinoma invaded the subserosal layer. No metaplastic mucosa was seen around the tumor. On December 7, 2015, the pathology of the bile duct biopsy from ERCP after recurrence identified only well-differentiated papillary adenocarcinoma and not the LCNEC component (Fig. 2c). The immunohistochemical staining results for MiNENs of the gallbladder in the cases are shown in Table 1.

**Table 1:** Immunohistochemical staining findings in two cases of gallbladder MiNENs

Antibody	Case 1		Case 2	
	LCNEC	PAC	LCNEC	PAC
Syn	diffusely strong positive	–	diffusely strong positive	–
CgA	weakly positive	–	diffusely strong positive	–
CEA	–	positive	–	positive
AE1/AE3	spotted weakly positive	diffusely strong positive	diffusely strong positive	diffusely strong positive
LCA	–	–	–	–
CD117	–	–	–	–
CD34	–	–	–	–
CK20	–	–	–	–
CK7	–	diffusely positive	–	diffusely positive
CDX2	–	–	–	–
P53	overexpression	overexpression	–	–
Ki67	80%	40%	80%	60%

*PAC* Papillary adenocarcinoma, *Syn* Synaptophysin, *CgA* Chromogranin A, overexpression: > 80%; −: Null

#### Case two

**Fig. 3:**
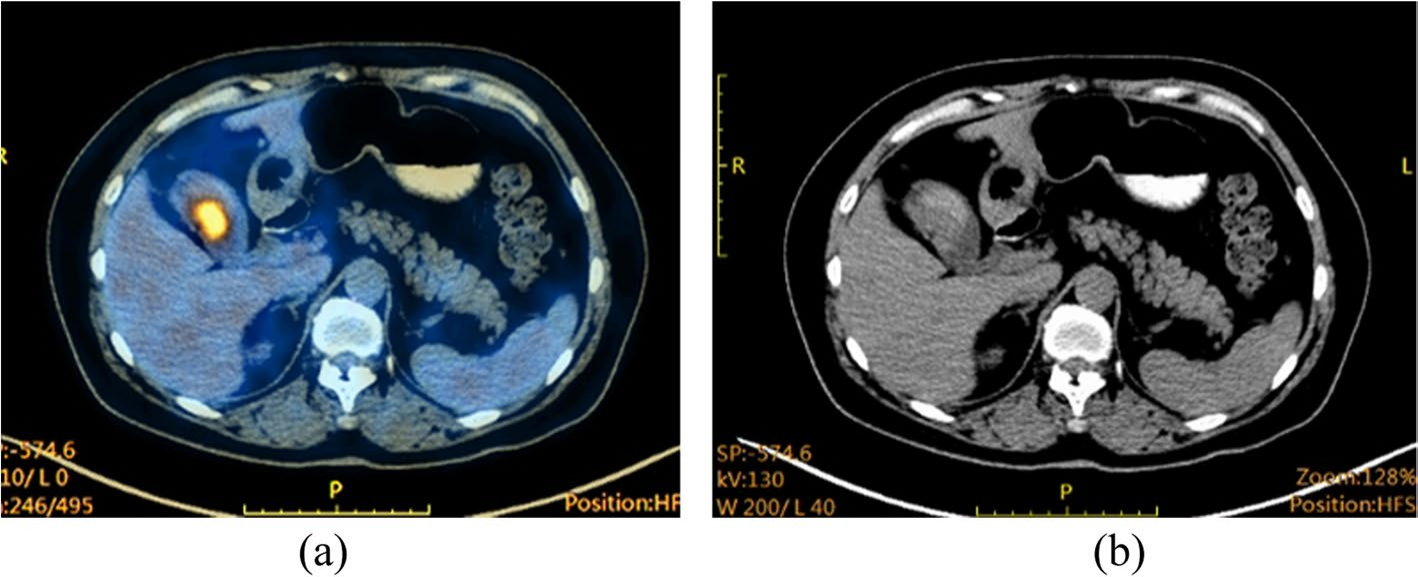
Case 2: ^18^FDG-PET/CT examination. **a** An indistinct hypointense mass and scattered slightly hyperdense calcified shadow in the gallbladder were observed. **b** FDG accumulated in the gallbladder mass

A 64-year-old female patient presented to our hospital on May 2, 2020, with a 1-week history of epigastric pain, nausea, and vomiting. Physical examination was only notable for localized abdominal tenderness. Preoperatively, all laboratory tests, including levels of tumor markers CEA, CA19–9, and NSE were normal. Ultrasonography revealed a wide-base nodular projection in the gallbladder, and further imaging studies including magnetic resonance cholangiopancreatography (MRCP), CT, and ^18^F-fluorodeoxyglucose-(^18^FDG)-positron emission tomography (^18^FDG-PET)/CT demonstrated a hypointense mass with a scattered, mildly calcified shadow in the gallbladder (Fig. 3a) and abnormal FDG accumulation in the mass (Fig. 3b), respectively, all of which suggested gallbladder cancer. Thus, the patient underwent en bloc cholecystectomy with hepatoduodenal ligament lymph node dissection.

**Fig. 4:**
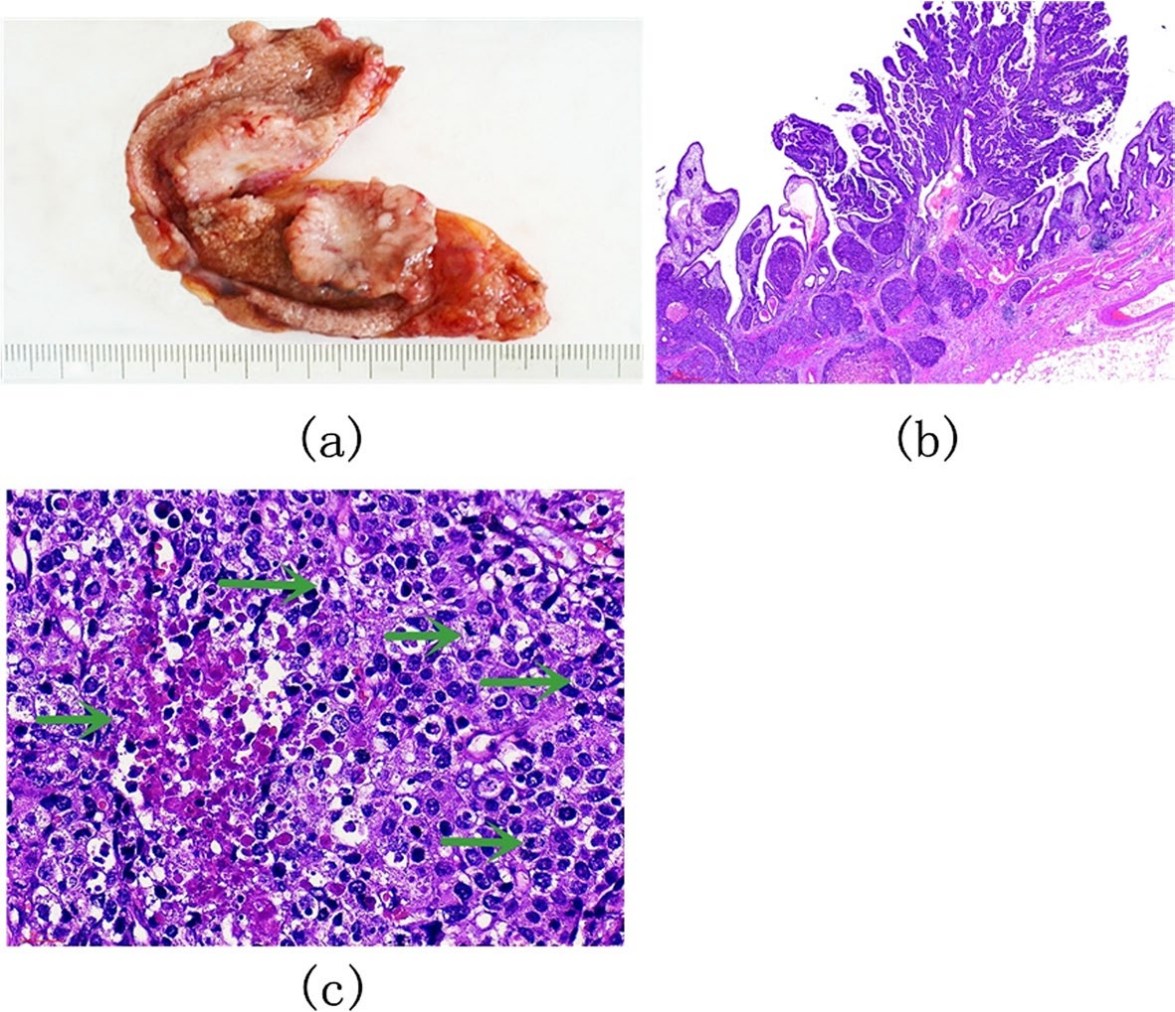
Case 2: Gross pathological findings of the resected gallbladder and histological findings of MiNEN in the gallbladder, by hematoxylin and eosin (HE) staining. **a** The body of the gallbladder was a grayish-white type Isp mass. **b** Two components, LCNEC (lower left, show multiple irregular organoid nests) and moderately differentiated papillary adenocarcinoma (upper right), were present, showing a mixed transitional section between the two different tissue components. Magnification, × 100. **c** Pleomorphic large cells with round to oval densely stained nuclei, visible nucleoli, coarse chromatin, abundant eosinophilic cytoplasm, high mitotic index (arrowhead), and a patchy necrosis in the center of the nests were revealed, × 400

In this case, a hard semipedunculated nodule with the size of 25 mm × 25 mm (Fig. 4a) was observed in the body of the gallbladder without gallstones. This neoplasm contained two cellular components with a composition ratio matching that of case 1 and was more significantly distributed in an organoid nest with a mixed transitional zone (Fig. 4b). A high mitotic rate (35 mitoses/2mm^2^) was observed, and the microscopic findings for LCNEC are shown in Fig. 4c. Moreover, LCNEC invaded the subserosal layer, and cancer emboli were observed in both blood vessels and lymphatic vessels. Metastasis in the regional lymph nodes was found predominantly with LCNEC components. The immunohistochemical staining results for MiNENs of the gallbladder, in this case, are shown in Table 1.

**Fig. 5:**
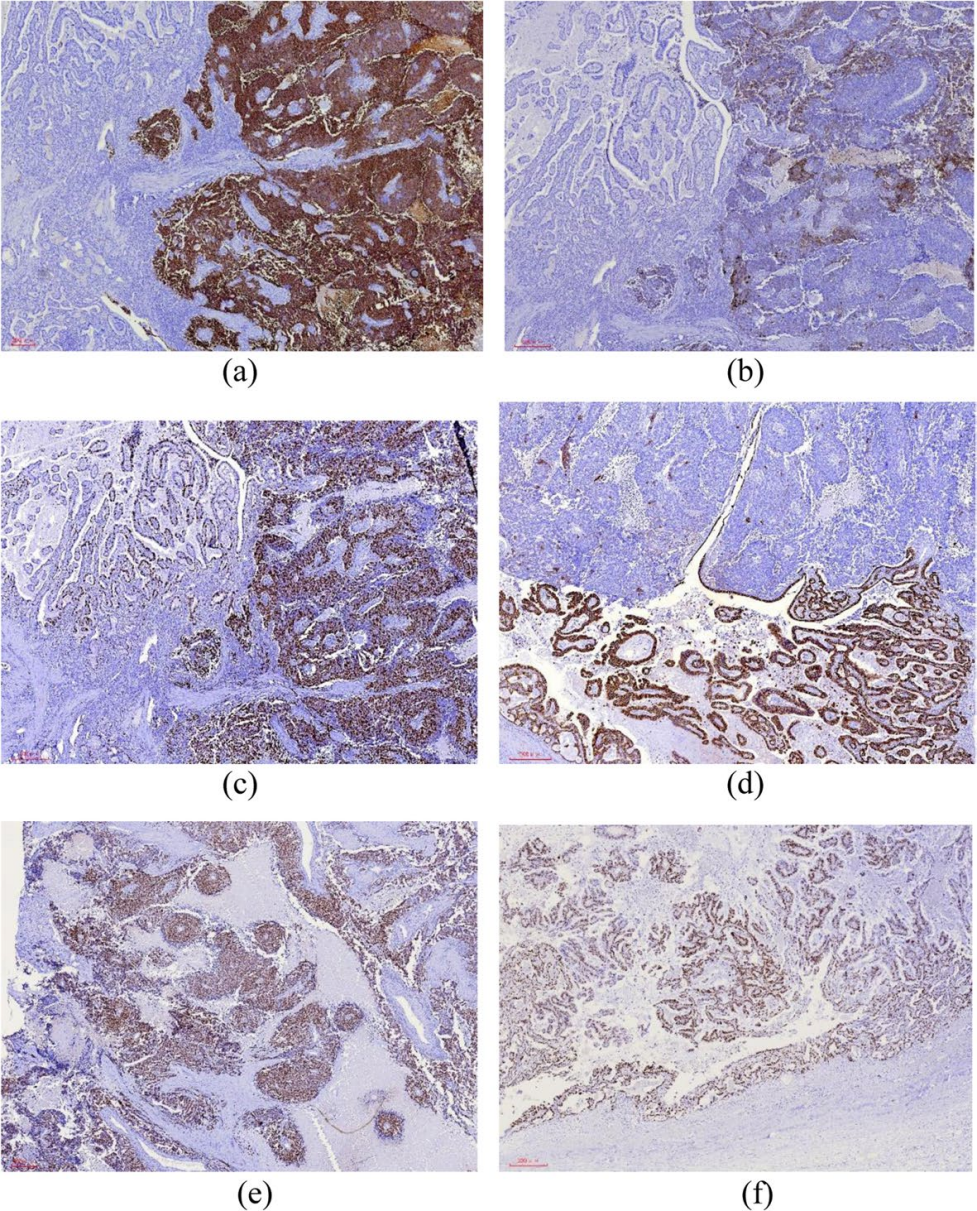
Case 1: Immunohistochemical staining findings for LCNEC and adenocarcinoma. **a** Syn staining was diffuse and strongly positive in LCNEC and negative in adenocarcinoma. Magnification, × 40. **b** Staining for CgA was weakly positive in LCNEC and negative in adenocarcinoma area. Magnification, × 40. **c** High Ki67 proliferation index was found in LCNEC and the adenocarcinoma component. Magnification, × 40. **d** AE1/AE3 staining was strongly positive in adenocarcinoma, and punctate weak positive staining was observed in LCNEC. Magnification, × 40. **e** *TP*53 staining showed overexpression in the LCNEC component (left) and in the adenocarcinoma component (upper right). Magnification, × 40. **f** *TP*53 staining also showed overexpression in the adenocarcinoma component (> 80%). Magnification, × 40

**Fig. 6:**
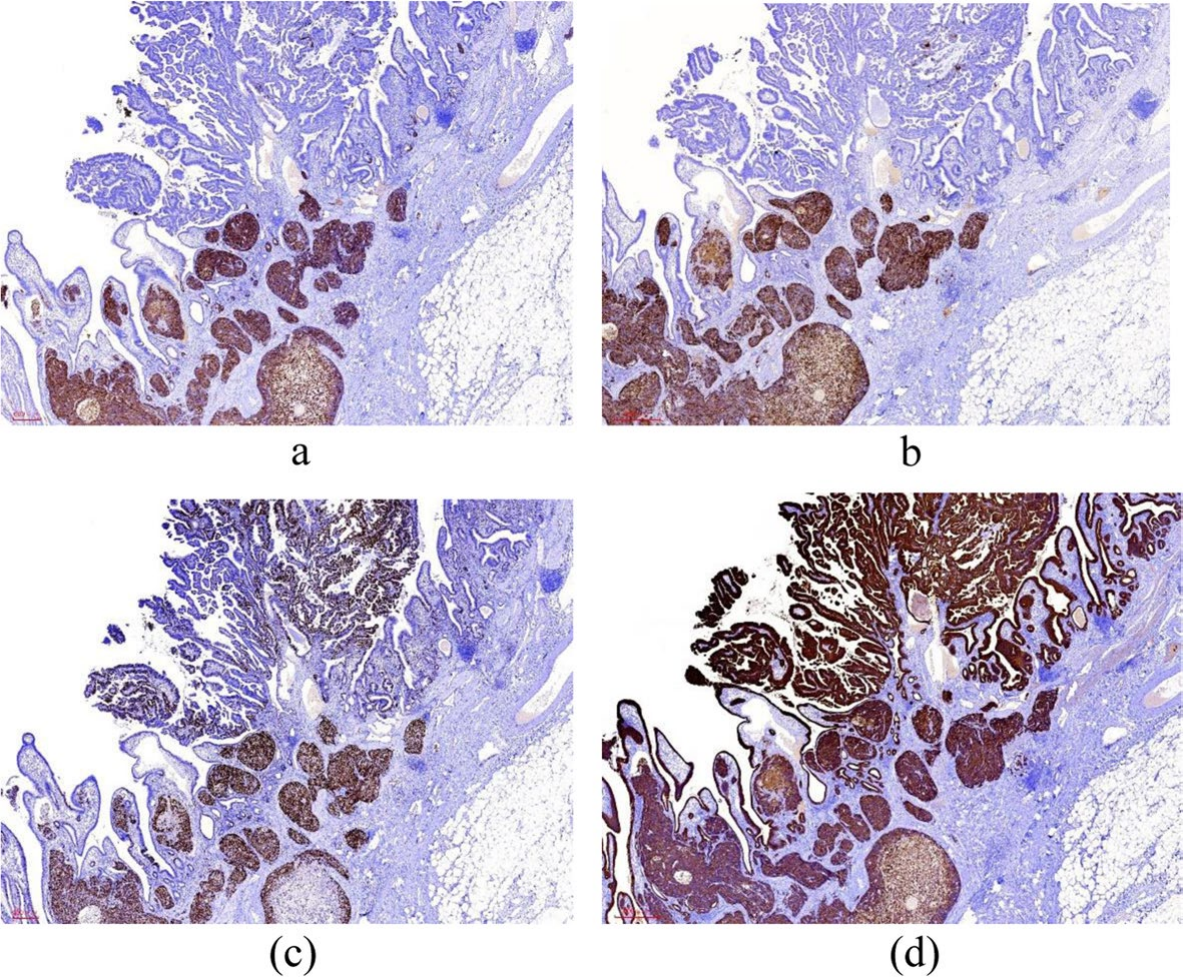
Case 2: Immunohistochemical staining findings for LCNEC and adenocarcinoma. **a** Syn staining was diffuse and strongly positive in LCNEC, and negative in adenocarcinoma. Magnification, × 20. **b** CgA staining was diffuse and strongly positive in LCNEC, and negative in adenocarcinoma. Magnification, × 20. **c** A high Ki67 proliferation index was identified in the LCNEC and the adenocarcinoma component. Magnification, × 20. **d** AE1/AE3 staining was strongly positive in both adenocarcinoma and the LCNEC component. Magnification, × 20

The immunohistochemical staining for synaptophysin (Syn), chromogranin A (CgA), AE1/AE3, tumor protein 53 *(TP53),* and Ki67 in both cases is shown in Figs. 5 and 6, respectively.

### Literature review

We found 72 case reports of gallbladder MiNENs in the literature, and along with the two cases presented above, proceeded with this review (Table 2). In our statistical analyses, the categorical variables were expressed as numbers and percentages, and the continuous variables were expressed as medians and ranges. Median survival outcomes were estimated by applying Kaplan–Meier analysis; moreover, the log-rank test was used to evaluate differences between groups. All data were analyzed using SAS9.4 satistical software.

**Table 2:** Summary of previously reported cases of mixed neuroendocrine-non-neuroendocrine neoplasms of the gallbladder

Case No.	Author [ref.]	Year	Age (yr)/ sex	Tumor size (mm)	Invasion depth	Metastasis Lymph node Liver		Ki-67(%) / mitoses (/2mm^2^)	NEN component	no-NEN component	Surgery and/or chemotherapy	Outcome (months)
1	Wisniewski et al. [5, 6]	1972	51/F	Large	hinf	–	+	NM	NEC	AC	Cholecystectomy with L	Death (NM)
2	Ito et al. [5, 6]	1980	75/F	60 × 35 × 20	NM	–	–	NM	NEC	Undifferentiated carcinoma	Cholecystectomy	Death (13)
3	Wada et al. [7]	1983	56/M	55 × 40 × 28	ss	–	–	NM/rarely	NET	Papillary AC	Cholecystectomy with RL	Death (16)
4	Muto et al. [8]	1984	80/M	10 × 7 × 7	ss	–	–	NM	NET, goblet cell adenocarcinoid	AC	Cholecystectomy with hepatic bed resection and RL	Survival (24)
5	Masuo et al. [5]	1984	74/M	NM	NM	–	+	NM	NEC	AC	Autopsy	Death (3)
6	Kotake et al. [5, 6]	1984	47/F	15 × 10	ss	+	+	NM	NEC	AC	Cholecystectomy	Death (17)
7	Haga et al. [5]	1988	79/M	35	hinf	–	–	NM	NEC	AC	Cholecystectomy with hepatic bed resection	Death (10)
8	Kurosaka et al. [5]	1988	46/F	40	ss	+	+	NM	NEC	AC	Cholecystectomy with hepatic bed resection	Survival (4)
9	Adachi et al. [9]	1988	69/F	55 × 30	hinf	–	–	NM	NEC	AC	Cholecystectomy with hepatic bed resection	Survival (12)
10	Yamamoto [6]	1989	76/F	25 × 25	ss	–	–	NM/common	NEC	Well-differentiated AC	Cholecystectomy	RF (93)
11		1989	77/F	Small polypoid	ss	–	–	NM/many	NEC	AC	Cholecystectomy	RF (17)
12	Fish et al. [10]	1990	77/F	110 × 70 × 30	T3	NM	–	NM/18	NEC	Moderately differentiated AC	Cholecystectomy with omentum resection	NM
13	Ohno et al. [5]	1991	63/F	140	ss	+	+	NM	NEC	AC	Cholecystectomy with hepatic bed resection	Death (9)
14	Cavazzana [11]	1991	71/F	50 × 30 × 30	T3	+	+	NM	SCNEC	Well-differentiated AC	Cholecystectomy with RL	Death (4)
15	Duan et al. [52]	1991	70/M	10	ss	+	+	NM	SCNEC	AC	Autopsy	Death (1)
16	Lida [12]	1992	62/F	65 X 30	H’inf	+	+	NM/frequently	SCNEC	AC, squamous cell carcinoma	Cholecystectomy with L	Death (5)
17	Ohmori et al. [13]	1993	78/F	40 × 30	T3	N2	M1	NM/frequently	NEC	AC	Autopsy	Death (1/4)
18	Murayama et al. [5]	1997	68/F	20	se	+	+	NM	NEC	AC	cholecystectomy	Death (2)
19	Yokoyama et al. [5]	1998	72/M	25	hinf	+	+	NM	NEC	AC	Cholecystectomy with hepatic bed resection	Survival (7)
20	Kamisawa et al. [5]	1998	48/F	50	hinf	+	+	NM	NEC	AC	Autopsy	NM
21	Furukawa et al. [5]	1998	68/M	35	hinf	–	–	NM	NEC	AC	Cholecystectomy with hepatic bed resection	Survival (18)
22	Moskal et al. [14]	1999	69/F	NM	T3	N2	M0	NM	SCNEC	Poorly differentiated AC	Chemotherapy, ERC	Death (44)
23		1999	40/M	NM	T2	N1	M0	NM	SCNEC	Moderately differentiated AC	ERC, chemotherapy	Survival (189)
24		1999	71/F	NM	T2	N2	MI	NM	SCNEC	Poorly differentiated AC	Palliative surgery, chemotherapy	Death (13)
25	Papotti et al. [15]	2000	50/M	Thickened GB wall	ss	–	–	50/> 20	LCNEC	AC	Cholecystectomy	RF (12)
26	Sakaki et al. [16]	2000	79/F	33 × 20 × 16	m	–	–	NM	SCNEC	AC	Cholecystectomy	RF (8)
27	Eriguchi et al. [5]	2000	81/F	26 × 16	ss	–	–	NM	NEC	Papillary AC, signet-ring cell carcinoma	Cholecystectomy	RF (8)
28	Yannakou [17]	2001	72/F	70 × 62 × 16	hinf	+	–	NM/high rate.	NEC	Well-differentiated AC	Radical cholecystectomy	Death (2)
29	Maitra et al. [18]	2001	85/F	40	mp	–	+	NM	SCNEC	AC	NM	Survival (13)
30		2001	77/F	28	T3	+	+	NM	SCNEC	AC	NM	Survival (25)
31		2001	73/F	25	mp	+	–	NM	SCNEC	AC	NM	Survival (7)
32		2001	82/M	10	mp	–	+	NM	SCNEC	AC, squamous cell carcinoma	NM	Survival (8)
33	Piana et al. [19]	2002	66/F	18	SS	–	–	NM/higher	SCNEC	Clear cell AC	Cholecystectomy, chemotherapy	Death (36)
34	Wakabayashi [20]	2003	71/F	100	Se	–	–	NM	NEC	AC, squamous cell carcinoma	extended liver resection	Survival (36)
35	Okamoto et al. [21]	2003	70/M	37 × 22	T3	+	+	NM	SCNEC	Papillary AC	Chemotherapy, cholecystectomy with L	Survival (0.5)
36	Koea et al. [22]	2004	68/F	MCN	se	+	–	NM	NEC	AC	Palliative surgery, chemotherapy	Death (6)
37	Mori et al. [20]	2005	70/F	36 × 15	ss	+	–	NM	NEC	AC, squamous cell carcinoma	Cholecystectomy with hepatic bed resection	Survival (32)
38	Shimizu et al. [23]	2006	58/M	150 × 90 × 120	hinf	NM	–	NM	SCNEC	AC	Cholecystectomy with hepatic trisegmentectomy	Death (4)
39	Noske [24]	2006	81/F	50 × 35 × 30	T3	N1	M1	NM	LCNEC	Adenosquamous carcinoma	Palliative surgery	NM
40	Tsuchiya et al. [25]	2006	36/F	10 × 8	ss	–	–	NM	NEC	Papillary AC	ERC	RF (12)
41	Sośnic and Sośnic [26]	2006	56/F	Thickened GB wall	hinf	–	–	NM	NEC	Papillary AC	Cholecystectomy with biliary-enteric anastomosis	Survival (0.3)
42	Hashimoto [27]	2007	55/F	18 × 12 × 5	ss	–	–	NM	NEC	AC, mucinous AC	Radical cholecystectomy	Survival (18)
43	Oshiro et al. [28]	2008	55/F	49 × 45	ss	–	–	73.3/NM 62.5/NM	LCNEC SCNEC	AC	ERC	RF (20)
44	Iype et al. [29]	2009	85/M	14 × 15	se	NM	+	NM	LCNEC	AC	Cholecystectomy, chemotherapy	Death (21)
45	Taniguchi et al. [30]	2009	62/M	100	T4	+	–	NM	SCNEC	AC	Chemotherapy, autopsy	Death (8)
46	Sato et al. [31]	2010	68/F	35	hinf	+	–	72/> 50	LCNEC	Well-differentiated AC	cholecystectomy with L	RF (12)
47	Kim et al. [32]	2011	48/F	95× 93 × 65	T3	+	–	NM	SCNEC	Moderately differentiated AC	ERC and chemotherapy	RF (18)
48	Paniz Monodolfi [33]	2011	48/F	35 × 33 × 24	hinf	+	+	NM/> 20	LCNEC	Papillary AC	Cholecystectomy with hepatic bed resection	NM
49	Harada et al. [34]	2012	70/F	35 × 25	hinf	+	–	12.3/59	SCNEC	Well-differentiated AC	NM	NM
50		2012	70/F	45 × 10	se	–	–	32.3/137	LCNEC	Well-differentiated papillary AC	NM	NM
51		2012	70/F	45 × 25	T3	–	–	0.5/4	NET G2	Well-differentiated AC	NM	NM
52		2012	60/F	15 × 15	ss	+	–	28.5/95	SCNEC	Well-differentiated papillary AC	NM	NM
53		2012	50/F	150 × 120	hinf	+	–	15.1/42	LCNEC	Well-differentiated AC	NM	NM
54	Song et al. [35]	2012	55/F	7 0 × 30 × 20	T3	–	–	> 80/> 20	SCNEC	Moderately differentiated AC	Chemotherapy, radical cholecystectomy	RF (7)
55	Rastogi et al. [36]	2012	48/F	Thickened GB wall	T3	–	–	NM	NEC	AC	Cholecystectomy with hepatic bed resection and L	NM
56	Fujii et al. [37]	2012	72/F	100	T2b	N2	M1	28/NM	SCNEC	AC	Chemotherapy, autopsy	Death (2)
57	Russo et al. [38]	2012	59/F	45 × 40	hinf	+	+	NM/40	LCNEC	Mucinous carcinoma	Cholecystectomy with L	Survival (24)
58	Al-Brahim [39]	2013	45/M	57 × 55 × 51	T3	+	+	> 95/50	LCNEC	AC	Cholecystectomy, chemotherapy	NM
59	Shintaku [40]	2013	80/M	82 × 53 × 50	In situ	–	–	18.7/6.2	NET G2	Well-differentiated AC, squamous cell carcinoma	Cholecystectomy with RL	RF (8)
60	Abe et al. [20]	2013	81/F	20 × 40	ss	+	–	NM	NEC	AC, squamous cell carcinoma	Cholecystectomy with hepatic bed resection and RL	Survival (48)
61	Chen et al. [41]	2014	34/F	40	T3	+	–	> 50/NM	NEC	AC	Cholecystectomy with hepatic bed resection and RL	Survival (4)
62	Meguro [42]	2014	54/F	90 × 60	T2	–	–	80/NM	LCNEC	Poorly differentiated AC (ICPN)	ERC	RF (24)
63	Chatterjee et al. [43]	2014	73/F	15 × 6 × 6	m	–	–	NM/> 60	SCNEC	Moderately differentiated papillary AC	Cholecystectomy, chemoradiation	Survival (45)
64	Liu et al. [44]	2015	63/F	20	T2a	–	–	> 80/NM	LCNEC	AC	Radical cholecystectomy	RF (12)
65	Acosta et al. [45]	2015	55/F	35 × 24 × 12	se	+	–	NM/27	LCNEC	Well-differentiated AC	Cholecystectomy	NM
66	Kamboj et al. [46]	2015	65/F	NM	T3	–	+	NM	NEC	AC	Biopsy	Survival (2)
67	Azad et al. [47]	2015	62/F	20 × 20	se	–	–	15/NM	NEC	Moderately differentiated AC	Radical cholecystectomy	RF (24)
68	Jung et al. [48]	2018	54/F	43 × 40	T3	+	+	NM/33	LCNEC	Adenosquamous carcinoma	Radical cholecystectomy, chemotherapy	Death (13)
69	Lin et al. [49]	2018	43/F	74× 56	T3	–	–	85/NM	SCNEC	Poorly differentiated AC	Radical cholecystectomy, chemotherapy	Survival (21)
70	Ines et al. [3]	2019	74/F	61	se	–	–	95/83	LCNEC	Well-differentiated AC	Cholecystectomy	Survival (7)
71	Skalický et al. [50]	2019	56/F	150	T4	+	+	70/64	SCNEC	AC	Cholecystectomy with L, chemotherapy	Survival (13)
72	Sciarra et al. [51]	2020	66/F	95	m	–	–	NM	LCNEC	AC, ICPN	Cholecystectomy with hepatic bed resection and RL	NM
73	Present		70/F	70 × 50	mp	+	–	80/> 60	LCNEC	Well-differentiated papillary AC	Cholecystectomy	Death (30)
74	Present		64/F	25 × 25	ss	+	–	80/> 60	LCNEC	Well-differentiated papillary AC	Cholecystectomy with hepatic bed resection and RL	Survival (12)

*NEN* Neuroendocrine neoplasm, *M* Male, *F* Female, *NM* Not mentioned, *NEC* neuroendocrine carcinoma, *AC* Adenocarcinoma, *NET* Neuroendocrine tumor, *RF* recurrence-free, *SCNEC* Small cell neuroendocrine carcinoma, *ERC* Extended radical cholecystectomy, *LCNEC* Large cell neuroendocrine carcinoma, *MCN* Multilocular cystic neoplasm, *GB* Gallbladder, *G2* Grade 2, *ICPN* Intracholecystic papillary neoplasm, *m* Mucosal layer, *mp* Muscle propria, *ss* Subserosal invasionse: tumor penetrated the serosa without invasion of adjacent structures, *hinf* Hepatic infiltration; cholecystectomy with RL: cholecystectomy with the cleaning of the regional lymph nodes; cholecystectomy with L: cholecystectomy with segmental liver resection

Demographically, the 74 patients had a mean age of 64.5 years, ranging from 36 to 85 years, with a ratio of male to female patients of 0.22. Clinically, more than two-thirds of patients presented with right upper quadrant or epigastric pain or discomfort (*n* = 34, 68%), and just over one-half were found to also have gallstones (*n* = 32, 51.6%). A few patients developed obstructive jaundice and weight loss, but some were asymptomatic. Preoperatively, enhanced CT and MR images showed enhancement of a homogeneous irregular mass as a high-intensity tumor, and ^18^FDG-PET/CT could detect accumulation of ^18^FDG in a mass or thickened gallbladder wall for poorly differentiated NECs. For cases in which difficulty occurred in establishing the diagnosis, ultrasound or CT- and endoscopic ultrasonography (EUS)-guided biopsy had diagnostic value. Tumor marker expression was not checked for all patients preoperatively, and among the 74 patients, the CEA level was only examined in 14 patients, of which five (35.7%) had an elevated CEA level in the range of 8 to 43 ng/ml (mean 22.6 ng/ml, normal < 5 ng/ml). The CA19–9 concentration was elevated in 11 of 20 patients (55.0%) tested, ranging from 73 to 728 U/ml (mean 215.3 U/ml, normal < 37 U/ml). An increase in AFP was found in 2 of 6 cases for which AFP was included in the work-up (157,428 ng/ml and 55.2 ng/ml).

Therapeutically, of the 74 patients with gallbladder MiNENs, 58 were treated surgically (78.4%), including 14 cases treated by simple cholecystectomy, 9 cases treated by cholecystectomy with gallbladder fossa liver tissue or liver bed wedge resection, 7 cases treated by cholecystectomy plus hepatectomy, 3 cases treated by cholecystectomy with regional lymph node dissection, 15 cases treated by en bloc cholecystectomy with hilar lymph node dissection (Glenn operation) or hepatectomy with hilar lymph node dissection, 6 cases treated by extended radical cholecystectomy (ERC) resection or extended to hepatopancreaticoduodenectomy, and 4 cases treated by palliative operations (Table 2).

Among the neuroendocrine components of MiNENs in the gallbladder, NEC without specified pathological subclassification (NSNEC) was the most common (*n* = 28, 37.8%), followed by small cell neuroendocrine carcinomas (SCNEC, *n* = 24, 32.4%), LCNEC (*n* = 18, 24.3%), and neuroendocrine tumours(NET) (*n* = 4, 5.4%). The non-neuroendocrine component, predominantly, was adenocarcinoma only, but in 14.9% of patients (*n* = 11), two or more non-neuroendocrine components co-existed (*n* = 9), mainly adenocarcinoma with squamous cell carcinoma (Table 2), or two synchronous neuroendocrine components were present (*n* = 2). The vast majority (*n* = 64, 92.8%) had a mass with a nodular, giant, or polypoid pattern. The mass sizes were reported in 63 case reports and ranged from 10 to 150 mm (mean, 50.8 ± 36.1 mm). Most masses showed a sessile (type Is) or semipedunculated (type Isp) morphology, and a few (4.8%) were pedunculated (type Ip). A small number of patients were found to have non-mass MiNENs (*n* = 5, 7.3%), including 4 cases of localized or diffuse thickening of the gallbladder wall and one case of the multilocular cystic tumor (MCN).

The histological features of vascular invasion have been documented in the literature. For lymphovascular infiltration, in a case series with 13 cases, four patients had lymphatic invasion, while in another 15 cases reports, 10 cases had vascular invasion, of which only one had liver metastasis. Because the number of patients was low and there was no endpoint time in the group without lymphatic or vascular invasion, the median survival time could not be calculated.

**Fig. 7:**
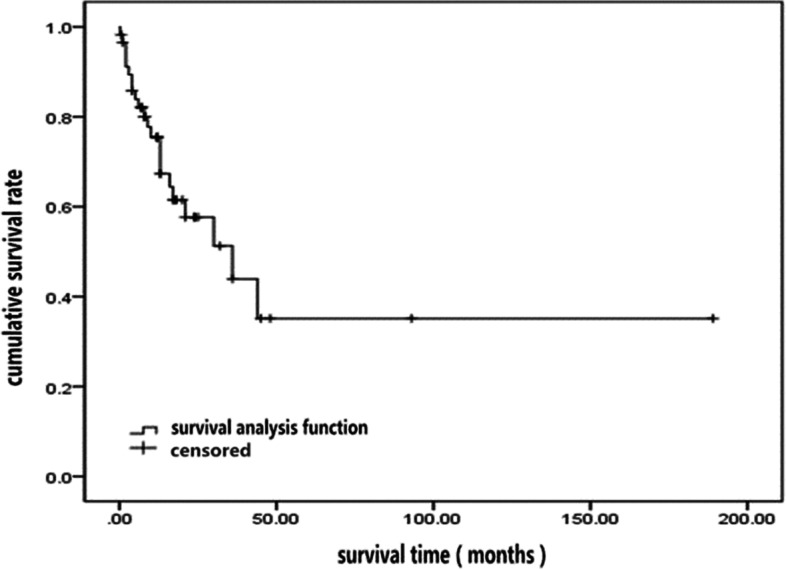
Median survival time of patients with gallbladder MiNENs (*n* = 59)

Moreover, nearly half of the patients had liver invasion with staging above T3 (*n* = 33, 45.8%), and more than half had regional lymph node metastasis (*n* = 37, 52.1%). One-third had liver metastasis (*n* = 26, 35.1%), and a few had metastasis of the bone, lung, skin, other abdominal organs (adrenal gland, pancreas), or peritoneal metastasis. Occasionally MiNENs in the gallbladder metastasized to the eyeball or femoral head. The median survival time of MiNEN patients (*n* = 59) was 36 ± 11.42 months (95% confidence interval (CI) 13.62 to 58.38 months; Fig. 7). Approximately one-fourth of cases received postoperative adjuvant chemotherapy (PAC) (*n* = 15, 25.9%) with a median survival time of 36 ± 15.46 months (95% CI, 5.70 to 66.30 months). In comparison, the median survival time of 43 patients who did not receive PAC was 30 months. Log-rank analysis was used to compare the survival times of patients who did or did not receive postoperative adjuvant chemotherapy, and the log-rank comparison statistic was 0.15 (*P* = 0.698), indicating the difference was not statistically significant.

## Discussion and conclusions

MiNEN of the gallbladder is an extremely rare disease that is more common in women than men with a male-to-female ratio of 0.22. The majority of patients with MiNENs presented with abdominal pain or discomfort or merely cholelithiasis-like symptoms in the early stage, and did not develop any symptom of carcinoid syndrome as the initial presentation, indicating carcinoid syndrome-causing chemicals might not be produced, or just sequestered in the biliary system in the early phase of the disease. Most gallbladder NiNENs formed a nodular or polypoid mass, which could develop into a large mass that invades adjacent organs such as the liver. A few cases were characterized by features of either localized or diffuse gallbladder wall thickening or degeneration due to tumor necrosis.

Histopathologically, the gallbladder MiNEN contains two tumor components of neuroendocrine and non-neuroendocrine type, with ≥30% of each component. Usually, the neuroendocrine component coexists with adenocarcinoma, but rarely is it found with other rare cancers or with two or more non-neuroendocrine components, such as squamous cell carcinoma, adenosquamous carcinoma, undifferentiated carcinoma, mucinous adenocarcinoma, signet ring cell carcinoma, sarcomatoid, osteosarcomatous and intracholecystic papillary neoplasm (ICPN), etc. [27, 51]. Also, two neuroendocrine components such as LCNEC and SCNEC can coexist with one adenocarcinoma [28]. The origin of MiNENs remains unclear since the normal gallbladder mucosa does not contain neuroendocrine cells, except for the gallbladder neck region [43]. Immunohistochemistry of gallbladder MiNENs in our case simultaneously revealed *TP53* overexpression and a high Ki67 proliferation index in LCNEC and adenocarcinoma of two different epithelial tumors, thereby suggesting that the two different components had the same molecular background [3, 51].

Moreover, the gallbladder NECs has two subclasses as SCNEC and LCNEC [4]. In this review of 74 cases of MiNENs of the gallbladder, NEC without specified pathology was the most common type (37.8%), followed by SCNEC (32.4%), then LCNEC (24.3%), and NETs (5.4%). The reason for the terminology of NEC rather that either SCNEC or LCNEC being more commonly used in many case reports might result from the adoption of the previous World Health Organization (WHO) classification of NENs of the digestive system. Furthermore, the current review found that patients with MiNENs of the gallbladder most likely had higher regional lymph node metastasis (50.7%) and hepatic metastasis rates (34.3%) compared with 15 and 17% of NEC of the gallbladder, respectively [4], suggesting that two co-exciting cancerous components may be one of the potential pathogenic mechanisms for enhanced invasiveness, even though some data reveal that the grade of the neuroendocrine component correlates with prognosis [23, 48]. Fifty-eight postoperative patients with gallbladder MiNENs had a short survival time of 36 months, although it seemed longer than the overall survival (25 months) of 754 patients with gallbladder NEC [4]. This difference in survival time might be affected by differences in the therapies applied.

Importantly, the size of NEC is not necessarily proportional to the metastatic potential, with the evidence indicating that even small primary NEC lesions also may infiltrate deeply or develop distant metastasis. In our review, three of four gallbladder MiNENs with a tumor size of 1 cm developed local invasion beyond the subserosal layer, and two (50%) led to liver metastasis and/or lymph node metastasis, one of which produced extensive metastasis to the liver, rectum, lung, adrenal gland, and pancreas before detection of the primary lesion of the gallbladder on ultrasound or CT examination [52]. In addition, of all seven T1 tumors in the review, except for one case of carcinoma in situ and two cases of mucosal cancer, three SCNEC cases and one LCNEC case with T1 stages were found to have lymph node or liver metastasis. Thus, MiNEN of the gallbladder may possess early metastatic potential.

It has been noted that the two different histological types of MiNEN of the gallbladder often metastasize separately [48]. In other words, the synchronous metastatic hepatic nodule is only composed of one component of MiNEN, while the metachronous metastatic hepatic nodules may be composed entirely of another component. However, this usually depends on the metastatic potential of each pathological component in the MiNEN [24, 48]. In our first case, the tumor metastasis and infiltration to the middle extrahepatic bile duct was found to be papillary adenocarcinoma without the LCNEC component. The survival of MiNEN patients mainly depends on NEC, which is closely related to lymph node and liver metastasis [25]; however, in case one, metastasis of the adenocarcinoma may have been the cause of death for this patient. Therefore, the malignancy of the two components of MiNENs may be separate determinants of the long-term prognosis.

Technically, the neuroendocrine components of MiNEN of the gallbladder can be determined by immunolabeling. Strong positive staining for Syn, CgA, NSE, somatostatin, etc. [46], and ultrastructural electron microscopy, even if a small number of neurosecretory granules (NSG) are found, all help the identification of neuroendocrine cells to establish the diagnosis of NEN [23]. Duan et al. [52] reported cases of coexistence of SCNEC and adenocarcinoma of the gallbladder, and the small cell carcinoma was noted with only weakly positive immunoexpression for NSE and a negative reaction to argentaffin staining or staining for other neurosecretory markers including CgA. However, electron microscopic examination, on the other hand, revealed NSG in the cytoplasm of some tumor cells, suggesting neuroendocrine tumors. Detection of serum NSE and CgA levels can also be used for diagnosis. The present two cases of MiNENs of the gallbladder showed positive expression of both Syn and CgA on immunohistochemical staining of LCNEC, and further histological analysis showed large and pleomorphic cells arranged in solid sheets or organoid nests. Moreover, the tumor cells had large-sized round to oval nuclei with visible vesicular nuclei, some visible prominent nucleoli, coarse chromatin, abundant cytoplasm, quick mitotic activity (exceeding 20 mitoses/2 mm^2^), a high Ki67 index of over 20%, and frequent large areas of necrosis, features consistent with the characteristics of LCNEC [4, 39, 44, 53].

Clinically, the diagnosis of MiNEN mainly relies on various imaging studies. Abdominal ultrasonography as a first imaging modality showed hypoechoic irregular solid nodules of heterogeneous internal echoes with increased blood flow that may be characteristic of NEC of the gallbladder. On contrast-enhanced CT scanning and MRI examination of the gallbladder, MiNENs appeared as an irregular mass, homogeneous, and strongly enhanced as high-intensity tumors [20]; however, the enhanced tumorous lesions could not be differentiated from gallbladder cancer [28]. In patients with localized thickening of the gallbladder wall, CT scanning without enhancement revealed a low-intensity thickening of the gallbladder wall or the appearance of debris [25], and CT with contrast showed heterogeneous ill-defined soft tissue enhancement along the gallbladder fossa. MRI showed local non-enhanced areas of altered signal intensity, indicating the presence of cystic degeneration [36]. The diagnosis has been difficult to establish for MiNENs of small size, with localized thickening of the gallbladder wall, cystic degeneration due to tumor epithelial cells secreting mucin [22] or necrosis, or the presence of multiple gallstones. Five cases from the literature [8, 15, 25, 36, 52], which had either negative primary gallbladder tumorous lesions or benign imaging findings, included three cases with 1-cm sized neoplasms only and two with localized thickening of the gallbladder wall including one case with both wall thickening and small cystic degeneration. Under such circumstances, PET-CT in one patient with thickened walls showed a heterogeneously enhanced mass with FDG accumulation (FDG-avid) in the gallbladder fossa, suggestive of gallbladder cancer [36].

The use of radionuclide ^18^FDG to diagnose MiNENs of the gallbladder has high sensitivity and specificity, but ^18^FDG-PET/CT can also lead to false-negative results for well-differentiated NENs [50]. ^18^FDG-PET/CT may highlight the accumulation of ^18^FDG in the mass for the gallbladder NEC with the effective clinical diagnosis, and therefore, has been useful for identifying the origin of lymph node metastases [54]. Case two in our case report showed intense FDG uptake in the gallbladder mass. Additionally, functional radiographical imaging such as somatostatin receptor (SSR) imaging with PET can be used to diagnose and differentiate NETs from gallbladder cancer. Since most NETs hold the characteristics of overexpression of SSR on the cell surfaces, radionuclide-labeled somatostatin analogs can tightly bind to SSR for receptor-dependent metabolic changes detected by PET-CT. For example, somatostatin receptor scintigraphy (SRS) uses ^111^In-octreotide for staging and diagnosing gallbladder NECs. PET-CT with ^68^Ga-DOTA-NOC as an alternative to SRS can show a hypermetabolic mass [46]. Also, this has the advantages of spatial resolution and better sensitivity and is a faster procedure.

For more obscure imaging findings, biopsy with histological examination should be the last resort. Fine needle aspiration (FNA) biopsy is usually performed under the guidance of either percutaneous ultrasound or CT [8, 14]. In addition, EUS-guided transmucosal FNA is another option [36], which can significantly improve the diagnostic sensitivity to 90% from 74% for EUS alone [35]. It should be emphasized that biopsy is only applied to confirm the diagnosis and not for early diagnosis.

The standard management of early-stage MiNENs of the gallbladder is the same as that for gallbladder cancer, involving radical cholecystectomy, that is, cholecystectomy with en bloc resection of the liver parenchyma surrounding the gallbladder bed and hepatoduodenal ligament lymphadenectomy, and liver segmentectomy is recommended for patients with locally advanced disease [50]. Additionally, simple cholecystectomy is also recommended for early-stage gallbladder NETs such as the T1N0 stage [35]. Pathological stage pT2 and localized liver invasion pT3 gallbladder cancers are suitable for extended radical cholecystectomy [55]. In the present review, among 58 cases of MiNENs of the gallbladder treated by surgical intervention, except for 14 cases of simple cholecystectomy and 4 cases of palliative surgery, the remaining 40 patients in the case reports all underwent radical resection with different ranges of resection according to the degree of tumor progression. Radical cholecystectomy including hepatic segmentectomy seems to improve the 5-year overall survival rate [35]. All six cases of gallbladder MiNENs in Table 2 that had a wide range of local infiltration were treated by extended radical cholecystectomy. Either hepatopancreaticoduodenectomy or pancreaticoduodenectomy was performed in four cases with hepatopancreatic metastasis and did improve the prognosis of these four patients. Although the number of cases was small and the approach could not be statistically compared with other methods, these cases showed that complete resection of the tumor tended to prolong survival [14, 25, 28, 32, 42], and for gallbladder NECs, patients with unresectable masses have a poor prognosis even when treated with chemotherapy and radiation therapy [44].

Neoadjuvant and adjuvant chemotherapies have been proposed as the initial management choice even for surgically resectable cases. Considerable evidence supports the effectiveness of platinum-based drug regimens in the treatment of SCNEC, and this treatment also may be suitable for LCNEC. However, no randomized clinical trials are showing superior efficacy compared with the alternative strategies used for non-neuroendocrine cancers [53], and solid clinical evidence remains lacking for the long-term survival benefit of the regimens. Despite all of this, surgical treatment with adjuvant chemotherapy has been advocated as the putative paradigm for NECs, and postoperative chemotherapy is recommended for advanced stages. However, because MiNEN is rarely sporadic in clinical practice, no general agreement has been reached regarding whether patients with MiNEN should receive chemotherapy, due to the fact that a poor overall response rate has been observed with drugs such as doxorubicin, 5-fluorouracil, cisplatin, and streptozocin alone or in combination [20]. Even though some studies claim that adjuvant chemotherapy may potentially improve the survival of NEC patients [35], a minority only received postoperative chemotherapy or multimodal therapy (21%) for gallbladder NETs, and 70% of patients with gallbladder NETs did not receive any additional therapy after surgery [4]. In the present review, only 15 of 58 patients (25.9%) with gallbladder MiNENs who received postoperative chemotherapy did not show any prolongation in their survival time.

Somatostatin analogs, as a new anti-NEN modality that possesses the effects of anti-tumor proliferation, inhibition of tumor angiogenesis, and promotion of tumor apoptosis, have been used to treat patients with confirmed somatostatin receptor expression on the surface of tumor cells through inhibition of the secretion of a variety of hormones by binding to the somatostatin receptors [35, 50]. Biologic therapies such as long-acting octreotide or lanreotide are able to prolong the overall survival of patients with metastatic mid-gut NEN and ameliorate their symptoms [35, 47]. Neoadjuvant chemotherapy combined with somatostatin successfully converted unresectable MiNEN cases to ones that could be treated by radical resection [35], indicating chemotherapy combined with somatostatin analogs might exert a therapeutic benefit for the long-term prognosis of MiNEN patients.

About one-third of patients had elevated CEA, while more than half of patients were found to have elevated CA19–9. In addition, AFP-producing gallbladder cancer is very rare. The pathological cause of elevated AFP in the gallbladder with NiNEN remains unclear; however, AFP-producing gallbladder cancer is prone to hepatic metastasis and has a poor prognosis [37].

This literature review carried a significant limitation. Since the cases from the literature were not consecutive, and the data extracted from the cases were heterogeneous, it could be impossible to conduct important studies like the prognosis study for long-term assessment of the patients with the disease. In general, such studies presumably require a stringent follow-up by our groups by sending out a questionnaire to each of the patients from the case reports, which could not be done in the reality.

In conclusion, about one-half of patients with MiNENs of the gallbladder, as an extremely rare disease with female predominance, mainly presented with the symptoms of cholelithiasis in the early stage. Preoperatively, the patients might be found to have lymph node metastasis and liver invasion, thus, contrast-enhanced CT, MRI, and ^18^FDG or ^68^Ga-DOTA-NOC PET-CT possessed superior value for establishing the diagnosis and planning the treatment choices for NENs. Besides these, either percutaneous or EUS-guided biopsy might also be an effective diagnostic alternative. Essentially, characteristic microscopic cell morphology-findings, Syn and/or CgA expression detected by immunohistochemical staining, NSGs observed by electron microscopy, and NEC and adenocarcinoma components each constituting ≥30% of a neoplasm provided evidence for the patho-histological diagnosis of gallbladder MiNEN. Therapeutically, extended radical cholecystectomy and either adjuvant or neoadjuvant chemotherapy combined with somatostatin analog treatment could be used to treat patients with advanced disease, however, a detailed prognosis analysis should be conducted before claiming the treatments could be beneficial for MiNENs.

## Data Availability

The datasets used and/or analyzed during the current study are available from the corresponding author on reasonable request.

## Abbreviations

MiNENs: Mixed neuroendocrine–non-neuroendocrine neoplasms
NEC: Neuroendocrine carcinoma
NSE: Neuron-specific enolase
CEA: Carcinoembryonic antigen
AFP: Alpha-fetoprotein
CT: Computed tomography
MRI: Magnetic resonance imaging
ERCP: Endoscopic retrograde cholangiopancreatography
LCNEC: large cell neuroendocrine carcinoma
MRCP: Magnetic resonance cholangiopancreatography
^18^FDG-PET: ^18^F-fluorodeoxy glucose-(^18^FDG)-positron emission tomography
Syn: Synaptophysin
CgA: Chromogranin A
*TP53*: Tumor protein 53
EUS: Endoscopic ultrasonography
ERC: Radical cholecystectomy
SCNEC: small cell neuroendocrine carcinoma
MCN: Multilocular cystic tumor
CI: Confidence interval
PAC: Postoperative adjuvant chemotherapy
ICPN: Intracholecystic papillary neoplasm
NEN: Neuroendocrine neoplasm
NET: Neuroendocrine tumor
WHO: World Health Organization
NSG: Neurosecretory granules
SSR: Somatostatin receptor
SRS: Somatostatin receptor scintigraphy
FNA: Fine-needle aspiration

## References

[1] Adsay NV, La RS (2019). Tumours of the gallbladder and extrahepatic bile duct. Digestive system tumours/WHO classification of tumours editorial board, 5th edition.

[2] Frizziero M, Chakrabarty B, Nagy B, Lamarca A, Hubner RA, Valle JW, et al. Mixed neuroendocrine non-neuroendocrine neoplasms: a systematic review of a controversial and underestimated diagnosis. J Clin Med. 2020;9(1):273. 10.3390/jcm9010273 PMC7019410 31963850

[3] Ines FMS, Anceno AL, Salamat RA, Navarro JN, Pua GL, Andal JJ (2019). Targeted sequencing of mixed neuroendocrine-non-neuroendocrine neoplasm of the gallbladder suggests a monoclonal origin: a case report. Philipp J Pathol.

[4] Ayabe RI, Wach M, Ruff S, Martin S, Diggs L, Wiemken T (2019). Primary gallbladder neuroendocrine tumors: insights into a rare histology using a large national database. Ann Surg Oncol.

[5] Eriguchi N, Aoyagi S, Noritomi T, Imamura M, Sato S, Fujiki K (2000). Adeno-endocrine cell carcinoma of the gallbladder. J Hepato-Biliary-Pancreat Surg.

[6] Yamamoto M, Nakajo S, Miyoshi N, Nakai S, Tahara E (1989). Endocrine cell carcinoma (carcinoid) of the gallbladder. Am J Surg Pathol.

[7] Wada A, Ishiguro S, Tateishi R, Ishikawa O, Matsui Y (1983). Carcinoid tumor of the gallbladder associated with adenocarcinoma. Cancer.

[8] Muto Y, Okamoto K, Uchimura M (1984). Composite tumor (ordinary adenocarcinoma, typical carcinoid, and goblet cell adenocarcinoid) of the gallbladder: a variety of composite tumor. Am J Gastroenterol.

[9] Adachi K, Fuji T, Nakata K, Noguchi T, Aibe T, Takemoto T (1988). Association of adenocarcinoma and endocrine cell carcinoma in the gallbladder--a case report. Nihon Shokakibyo Gakkai Zasshi.

[10] Fish DE, Al-Izzi M, George PP, Whitaker B (1990). Combined endocrine cell carcinoma and adenocarcinoma of the gallbladder. Histopathology.

[11] Cavazzana AO, Fassina AS, Tollot M, Ninfo V (1991). Small-cell carcinoma of gallbladder. An immunocytochemical and ultrastructural study. Pathol Res Pract.

[12] Iida Y, Tsutsumi Y (1992). Small cell (endocrine cell) carcinoma of the gallbladder with squamous and adenocarcinomatous components. Acta Pathol Jpn.

[13] Ohmori T, Furuya K, Okada K, Tabei R, Tao S (1993). Adenoendocrine cell carcinoma of the gallbladder: a histochemical and immunohistochemical study. Acta Pathol Jpn.

[14] Moskal TL, Zhang PJ, Nava HR (1999). Small cell carcinoma of the gallbladder. J Surg Oncol.

[15] Papotti M, Cassoni P, Sapino A, Passarino G, Krueger JE, Albores-Saavedra J (2000). Large cell neuroendocrine carcinoma of the gallbladder: report of two cases. Am J Surg Pathol.

[16] Sakaki M, Hirokawa M, Sano T, Horiguchi H, Wakatsuki S, Ogata S (2000). Gallbladder adenocarcinoma with florid neuroendocrine cell nests and extensive Paneth cell metaplasia. Endocr Pathol.

[17] Yannakou N, Rizos S, Parissi-Mathiou P, Smailis D, Charanioti S, Dervenis C (2001). Mixed (composite) glandular-endocrine cell carcinoma of the gallbladder. HPB (Oxford).

[18] Maitra A, Tascilar M, Hruban RH, Offerhaus GJ, Albores-Saavedra J (2001). Small cell carcinoma of the gallbladder: a clinicopathologic, immunohistochemical, and molecular pathology study of 12 cases. Am J Surg Pathol.

[19] Piana S, Cavazza A, Corrado S, Putrino I, Gardini G (2002). Combined small cell carcinoma and clear cell carcinoma of the gallbladder: report of a case and review of the literature. Pathol Res Pract.

[20] Abe T, Kajiyama K, Harimoto N, Gion T, Shirabe K, Nagaie T (2013). Composite adeno-endocrine carcinoma of the gallbladder with long-term survival. Int J Surg Case Rep.

[21] Okamoto H, Miura K, Ogawara T, Fujii H, Matsumoto Y (2003). Small-cell carcinoma manifesting systemic lymphadenopathy combined with adenocarcinoma in the gallbladder: aggressiveness and sensitivity to chemotherapy. J Gastroenterol.

[22] Koea J, MacCormack M, Findlay M, Ramsdorp R (2004). Adeno-endocrine cancer of the gallbladder. ANZ J Surg.

[23] Shimizu T, Tajiri T, Akimaru K, Arima Y, Yoshida H, Yokomuro S (2006). Combined neuroendocrine cell carcinoma and adenocarcinoma of the gallbladder: report of a case. J Nippon Med Sch.

[24] Noske A, Pahl S (2006). Combined adenosquamous and large-cell neuroendocrine carcinoma of the gallbladder. Virchows Arch.

[25] Tsuchiya A, Endo Y, Yazawa T, Saito A, Inoue N (2006). Adenoendocrine cell carcinoma of the gallbladder: report of a case. Surg Today.

[26] Sośnik H, Sośnik K (2006). Double cancer of the gallbladder--a case report. Pol J Pathol.

[27] Hashimoto M, Okuda C, Sakurai C, Seki K, Matsuda M, Nagao G (2007). Adenoendocrine cell carcinoma of the gallbladder: differentiation of the endocrine component. J Gastroenterol Hepatol.

[28] Oshiro H, Matsuo K, Mawatari H, Inayama Y, Yamanaka S, Nagahama K (2008). Mucin-producing gallbladder adenocarcinoma with focal small cell and large cell neuroendocrine differentiation associated with pancreaticobiliary maljunction. Pathol Int.

[29] Iype S, Mirza TA, Propper DJ, Bhattacharya S, Feakins RM, Kocher HM (2009). Neuroendocrine tumours of the gallbladder: three cases and a review of the literature. Postgrad Med J.

[30] Taniguchi H, Sakagami J, Suzuki N, Hasegawa H, Shinoda M, Tosa M (2009). Adenoendocrine cell carcinoma of the gallbladder clinically mimicking squamous cell carcinoma. Int J Clin Oncol.

[31] Sato K, Imai T, Shirota Y, Ueda Y, Katsuda S (2010). Combined large cell neuroendocrine carcinoma and adenocarcinoma of the gallbladder. Pathol Res Pract.

[32] Kim DI, Seo HI, Lee JY, Kim HS, Han KT (2011). Curative resection of combined neuroendocrine carcinoma and adenocarcinoma of the gallbladder. Tumori.

[33] Paniz Mondolfi AE, Slova D, Fan W, Attiyeh FF, Afthinos J, Reidy J (2011). Mixed adenoneuroendocrine carcinoma (MANEC) of the gallbladder: a possible stem cell tumor?. Pathol Int.

[34] Harada K, Sato Y, Ikeda H, Maylee H, Igarashi S, Okamura A (2012). Clinicopathologic study of mixed adenoneuroendocrine carcinomas of hepatobiliary organs. Virchows Arch.

[35] Song W, Chen W, Zhang S, Peng J, He Y (2012). Successful treatment of gallbladder mixed adenoneuroendocrine carcinoma with neo-adjuvant chemotherapy. Diagn Pathol.

[36] Rastogi A, Bihari C, Singh S, Deka P, Bhatia V, Sarin S (2012). Adenoendocrinecarcinoma of gallbladder in a patient with primary sclerosing cholangitis and ulcerative colitis. Trop Gastroenterol.

[37] Fujii H, Yamaguchi K, Ohnishi N, Sakamoto M, Ohkawara T, Sawa Y (2012). Adenoendocrine cell carcinoma of the gallbladder producing a high level of alpha-fetoprotein. Clin J Gastroenterol.

[38] Russo S, Russo F, Maiello FM, Paolini B, Carrabba A, De Gregorio A (2012). Biphasic large cell neuroendocrine carcinoma--pure mucinous carcinoma of the gallbladder (MANEC): a unique combination. Pathologica.

[39] Al-Brahim N, Albannai R (2013). Combined large cell neuroendocrine carcinoma and adenocarcinoma of the gallbladder. Endocr Pathol.

[40] Shintaku M, Kataoka K, Kawabata K (2013). Mixed adenoneuroendocrine carcinoma of the gallbladder with squamous cell carcinomatous and osteosarcomatous differentiation: report of a case. Pathol Int.

[41] Chen H, Shen YY, Ni XZ (2014). Two cases of neuroendocrine carcinoma of the gallbladder. World J Gastroenterol.

[42] Meguro Y, Fukushima N, Koizumi M, Kasahara N, Hydo M, Morishima K (2014). A case of mixed adenoneuroendocrine carcinoma of the gallbladder arising from an intracystic papillary neoplasm associated with pancreaticobiliary maljunction. Pathol Int.

[43] Chatterjee D, Wang H (2014). Mixed adenoneuroendocrine carcinoma arising in a papillary adenoma of gallbladder. Am J Cancer Case Rep.

[44] Liu W, Wang L, He XD, Feng C, Chang XY, Lu ZH (2015). Mixed large cell neuroendocrine carcinoma and adenocarcinoma of the gallbladder: a case report and brief review of the literature. World J Surg Oncol.

[45] Acosta AM, Hamedani FS, Kajdacsy-Balla A, Wiley EL (2015). Primary mixed Adenoneuroendocrine carcinoma of the gallbladder in a 55-year-old female patient: a case report and review of the literature. Int J Surg Pathol.

[46] Kamboj M, Gandhi JS, Gupta G, Sharma A, Pasricha S, Mehta A (2015). Neuroendocrine carcinoma of gall bladder: a series of 19 cases with review of literature. J Gastrointest Cancer.

[47] Azad S, Shukla D, Garg A, Negi SS, Malhotra V (2015). Mixed adenoneuroendocrine carcinoma of the gallbladder, histopathological features. Indian J Pathol Microbiol.

[48] Jung J, Chae YS, Kim CH, Lee Y, Lee JH, Kim DS (2018). Combined Adenosquamous and large cell neuroendocrine carcinoma of the gallbladder. J Pathol Transl Med.

[49] Lin YX, Jia QB, Fu YY, Cheng NS (2018). Mixed Adenoneuroendocrine carcinoma of the gallbladder. J Gastrointest Surg.

[50] Skalický A, Vištejnová L, Dubová M, Malkus T, Skalický T, Troup O (2019). Mixed neuroendocrine-non-neuroendocrine carcinoma of gallbladder: case report. World J Surg Oncol.

[51] Sciarra A, Missiaglia E, Trimech M, Melloul E, Brouland JP, Sempoux C (2020). Gallbladder mixed neuroendocrine-non-neuroendocrine neoplasm (MiNEN) arising in Intracholecystic papillary neoplasm: Clinicopathologic and molecular analysis of a case and review of the literature. Endocr Pathol.

[52] Duan HJ, Ishigame H, Ishii Z, Itoh N, Shigematsu H (1991). Small cell carcinoma of the gallbladder combined with adenocarcinoma. Acta Pathol Jpn.

[53] Klimstra D, Klöppel G, La Rosa S, Rindi G (2019). Classification of neuroendocrine neoplasms of the digestive system. WHO Classification of tumours, 5th Edition Digestive system tumours.

[54] Okuyama Y, Fukui A, Enoki Y, Morishita H, Yoshida N, Fujimoto S (2013). A large cell neuroendocrine carcinoma of the gall bladder: diagnosis with 18FDG-PET/CT-guided biliary cytology and treatment with combined chemotherapy achieved a long-term stable condition. Jpn J Clin Oncol.

[55] Shirai Y, Sakata J, Wakai T, Ohashi T, Hatakeyama K (2012). “Extended” radical cholecystectomy for gallbladder cancer: long-term outcomes, indications and limitations. World J Gastroenterol.

